# Allergens involved in Food-Dependent Exercise-Induced Anaphylaxis (FDEIA) – experience of a centre in the North of Portugal

**DOI:** 10.1186/2045-7022-5-S3-P56

**Published:** 2015-03-30

**Authors:** Ana Reis Ferreira, Maria João Sousa, Inês Lopes, José Pedro Moreira da Siva

**Affiliations:** 1 Centro Hospitalar Vila Nova de Gaia/Espinho, Vila Nova de Ga, Portugal

## Background

FDEIA is a rare but potentially fatal disease. In Japan, the most frequently involved allergen is Ω-5-gliadin (Tri a 19), a protein present in wheat. N*on-specific Lipid Transfer Proteins* (nsLTPs) were described as the predominantly involved allergens in Italy. The objective of this study was to evaluate the allergens involved in FDEIA in our Allergy Clinic.

## Methods

Cross-sectional study of the patients of our Food Allergy Unit with FDEIA symptoms. Patient data regarding demography, personal history of atopy and suspected food were collected. Skin prick tests (SPT) with commercial extracts and skin prick-to-prick tests (SPPT) were performed. Specific IgE (sIgE) (ImmunoCAP Thermofisher^®^) for the suspected food and potentially involved molecular allergens were determined.

## Results

FDEIA was diagnosed in 6 patients, 4 male, median age 39±12.51 years (19-55), all atopic. In 5 patients, the food ingested before the episode of anaphylaxis was wheat bread (concomitant with shrimp in one patient and tree nuts in another) and apple in 1 patient.

Of the 5 patients with prior ingestion of wheat, 4 had positive SPT for wheat. SPPT to wheat flour was positive in the 3 patients in which it was performed. SIgE for rTri a 19 was positive in the 3 patients who had ingested bread alone and in the one who ate shrimp concomitantly (median - 7.88 UK/l [2.29 -12.5]); in the last patient, allergy to shrimp was excluded by OCT.

The patient with prior ingestion of tree nuts presented positive SPT and SPPT to various tree nuts; sIgE to Cor a 8 was 1.55 UK/l.

The patient with prior ingestion of apple presented positive SPT and SPPT to several fruits and positive SPT to LTP extract; sIgE to Pru p 3 was 4.49 UK/l.

No patient reported anaphylaxis since the culprit foods' ingestion was avoided pre-exercise.

## Conclusion

In this study it was possible to identify Ω-5-gliadin in 4 patients and nsLTPs in 2 patients as the allergens involved in FDEIA. Molecular allergens were helpful in the definitive diagnosis, especially since severe reactions may be induced by food challenge followed by exercise (the gold standard diagnostic procedure) in patients with FDEIA.

## Consent

Written informed consent was obtained from patients for publication of this abstract and any accompanying images. A copy of the written consent is available for review by the Editor of this journal.

